# A Comprehensive Machine Learning Framework for the Exact Prediction of the Age of Onset in Familial and Sporadic Alzheimer’s Disease

**DOI:** 10.3390/diagnostics11050887

**Published:** 2021-05-17

**Authors:** Jorge I. Vélez, Luiggi A. Samper, Mauricio Arcos-Holzinger, Lady G. Espinosa, Mario A. Isaza-Ruget, Francisco Lopera, Mauricio Arcos-Burgos

**Affiliations:** 1 Department of Industrial Engineering, Universidad del Norte, Barranquilla 081007, Colombia; 2 Department of Public Health, Universidad del Norte, Barranquilla 081007, Colombia; luiggis@uninorte.edu.co; 3 Grupo de Investigación en Psiquiatría (GIPSI), Departamento de Psiquiatría, Instituto de Investigaciones Médicas, Facultad de Medicina, Universidad de Antioquia, Medellín 050010, Colombia; oscararcos98@gmail.com; 4 INPAC Research Group, Fundación Universitaria Sanitas, Bogotá 111321, Colombia; lgespinosaar@unisanitas.edu.co (L.G.E.); misaza@unisanitas.edu.co (M.A.I.-R.); 5 Neuroscience Research Group, University of Antioquia, Medellín 050010, Colombia; floperar@gmail.com

**Keywords:** age of onset, machine learning, Alzheimer’s disease, genetic isolates, *PSEN1*, predictive genomics, natural history

## Abstract

Machine learning (ML) algorithms are widely used to develop predictive frameworks. Accurate prediction of Alzheimer’s disease (AD) age of onset (ADAOO) is crucial to investigate potential treatments, follow-up, and therapeutic interventions. Although genetic and non-genetic factors affecting ADAOO were elucidated by other research groups and ours, the comprehensive and sequential application of ML to provide an exact estimation of the actual ADAOO, instead of a high-confidence-interval ADAOO that may fall, remains to be explored. Here, we assessed the performance of ML algorithms for predicting ADAOO using two AD cohorts with early-onset familial AD and with late-onset sporadic AD, combining genetic and demographic variables. Performance of ML algorithms was assessed using the root mean squared error (RMSE), the R-squared (*R*^2^), and the mean absolute error (MAE) with a 10-fold cross-validation procedure. For predicting ADAOO in familial AD, boosting-based ML algorithms performed the best. In the sporadic cohort, boosting-based ML algorithms performed best in the training data set, while regularization methods best performed for unseen data. ML algorithms represent a feasible alternative to accurately predict ADAOO with little human intervention. Future studies may include predicting the speed of cognitive decline in our cohorts using ML.

## 1. Introduction

Alzheimer’s disease (AD; OMIM 104300) is a neurodegenerative disorder characterized by progressive loss of neurological, mental, and cognitive functions, including memory, changes in judgment, behavior, and emotions [1,2,3,4]. AD is the most common cause of dementia and constitutes an increasing challenge due to society’s public health and economic costs [5,6,7,8]. As of 2016, ~44 million people had AD or related dementia worldwide [9]. Without new medicines to prevent, delay, or stop the disease, this figure is projected to dramatically increase to ~66 million dementia cases by 2030 and ~116 million by 2050 [10]. The financial burden associated with the disease was estimated to be USD 818 billion in 2015 worldwide [11,12].

AD neuropathological damage is characterized by extracellular deposits of the beta-amyloid (Aβ) peptide, the formation of intracellular neurofibrillary tangles of hyperphosphorylated tau protein (p-Tau), and the impairment of neurons and synaptic connections in the cerebral cortex and hippocampus, a key brain region involved in learning and memory processes and emotional control [1,13,14,15]. Genetically, AD is divided into familial AD (*f*AD), which accounts for <5% of AD cases and is caused by the presence of pathogenic and deleterious mutations harbored in major genes (segregating in a mendelian way) such as *APP*, *PSEN1*, *PSEN2* [9,16,17,18], *ADAM10*, *AKAP9*, *PICALM*, *PLD3*, *TREM2*, and *UNC5C* [19,20,21,22,23,24,25,26,27], and sporadic AD (*s*AD), without a clear mendelian pattern of segregation, which accounts for >90% of AD cases. In contrast to *f*AD, mutations in genes associated with *s*AD do not directly cause AD but confer susceptibility [28]. Although *f*AD and *s*AD forms are phenotypically similar, the age of onset (AOO) at which signs and/or symptoms of AD appear for the first time in cases of *f*AD is generally earlier than in cases of *s*AD, with important predictors of ADAOO in *s*AD correspond to several genetic variants of small effect [29,30]. Indeed, it is generally established that *f*AD cases have an AOO before 65 years (ranging from the early 30s to the late 70s), while the AOO in *s*AD generally starts after 65 years [31,32]. Considering that in *s*AD cases are diagnosed later and usually at the later stages of disease compared to familial cases [16,28], developing predictive models of ADAOO will open new possibilities for clinicians, patients, and family members [33,34,35,36,37,38].

Despite being suggested ~25 years ago as a valuable quantitative phenotype for monitoring AD natural history [39], ADAOO is one of the least studied phenotypes in the epidemiology of AD [39,40,41]. In fact, recent studies of our group and from other research groups showed that the natural history of AD might lead to the elucidation of new diagnostic, predictive, and therapeutic alternatives while considering interventions to delay the ADAOO [33,42,43,44].

For more than three decades, our group characterized clinically and genetically the world’s most extensive known pedigree with an aggressive form of AD caused by the E280A mutation in the *PSEN1* gene, often referred to as the Paisa mutation [39]. Parallelly, we have characterized other forms of *f*AD and individuals with *s*AD from the same community who share the same genetic background of the E280A pedigree [45,46]. To the best of our knowledge, our group pioneered the exposition of major genetic variants modifying ADAOO in *s*AD [35]. Instead of using a traditional approach where the risk of developing AD is assessed [39,41], we recruited individuals with *s*AD exhibiting ADAOO at the extremes of the AOO distribution in order to identify genetic variants responsible for the wide spread of AOO [33,35,42,43,44]. Genes harboring these variants play an essential role in cell proliferation, apoptotic and immune dysregulation processes, oligodendrocyte differentiation, protein degradation, neuron apoptosis, cholesterol metabolism, neurogenesis, and inflammatory and memory processes linked to AD [35,36].

Predictive models aim to determine the expected value of an outcome variable *Y* of interest based on a set of predictors *X* = (*X*_1_, *X*_2_, …, *X_p_*)*^T^*. Generally speaking, *Y* and *X* can be of any nature (i.e., binary, multinomial, ordinal, or continuous), and the selection of the best predictive model is based on some sort of error-related measure, such as the accuracy, the root mean squared error (RMSE) or the mean absolute error (MAE) [47,48]. Although some predictive models have recently been developed for AD [49,50,51,52], the outcome variable is not ADAOO and genetic variants are not included as predictors. We argue that genetic/genomic data will substantially improve AD diagnosis and mitigate the confounding effect of demographic and population structure data while increasing power [53,54].

Machine learning (ML) has attracted the research community’s attention for disclosing patterns, detecting objects, and developing predictive frameworks in several diseases [55,56]. AD is one of the most common mental health conditions studied via ML methods [57]. In fact, we and others showed that ML algorithms constitute a promising alternative for assessing AD diagnosis based on prospective clinical, image, and/or biomarker data [18,58,59,60,61,62,63,64]. Furthermore, ML algorithms have also proven to be a suitable alternative for the timely diagnosis of late-onset AD based on genetic variation [29,32,65], differentiate AD from other neurological disorders using noninvasive blood markers [65], and predict AD conversion in individuals with Mild Cognitive Impairment (MCI) [66,67]. Interestingly, optimization procedures for tuning the parameters of ML algorithms have been reported to increase the sensitivity, specificity, and accuracy of ML for AD diagnosis [68]. Other ML alternatives include the use of artificial intelligence (AI), namely deep learning (DL), assessing AD diagnosis and progression with brain radiological images [69,70]. Although these results are promising, their main limitation is that the predictive model provided either an estimate of the risk of an individual for developing AD or the range within which the ADAOO may fall with high confidence (i.e., early- or late-onset based on whether the ADAOO was before or after a threshold, respectively), but not an estimate of the actual ADAOO. Moreover, a comprehensive exploration of advanced ML algorithms for ADAOO prediction is yet to be conducted.

In this study, we comprehensively assess ML algorithms’ feasibility applied to *f*AD and *s*AD cohorts, with the overarching aims of (1) accurately predicting ADAOO and improving the scope and performance previously reached; and (2) expanding the possibilities of quantifying ADAOO in the clinical setting. Our results suggest that ML constitutes a feasible and easy-to-implement new methodology to predict ADAOO, especially in the clinical setting, while significantly overpowering our previous results and paving the way for new possibilities to define follow-up and counseling strategies for patients and their family members.

## 2. Materials and Methods

### 2.1. Subjects

#### 2.1.1. E280A Pedigree

We ascertained 71 patients from the 459 E280A *PSEN1* mutation carriers at the extremes of the ADAOO distribution (44 women [62%] and 27 men [38%]) [33,36]. Detailed clinical assessment and ascertainment procedures of this pedigree have been presented elsewhere [31,71,72,73].

#### 2.1.2. The Cohort of Sporadic Cases

Fifty-four individuals with *s*AD were included in this study (43 [80%] were women, and 11 [20%] men). Clinical, neurological, and neuropsychological assessment of *s*AD patients has been reported elsewhere [35]. ADAOO was determined during anamnesis with the information provided by patients or their families, with confirmation by several sources. Because some patients started their follow-up during MCI, ADAOO was defined during the follow-up stage based on Petersen’s criteria [74]. This strategy was recently proven to be highly accurate [75]. AD affection status was defined based on the DSM-IV criteria [76].

### 2.2. Variants Associated with ADAOO

We previously studied the association of common exonic functional variants (CEFVs) with ADAOO (Table 1) [35,36] using single- and multi-locus linear mixed-effects models [77] and recursive partitioning ML algorithms [36]. These variants were found to delay ADOO up to 17 years in carriers of the E280A *PSEN1* mutation and accelerate it up to ~14 years in individuals with *s*AD [35,36].

### 2.3. ADAOO Prediction Using ML

Predictive models of ADAOO were constructed with ML algorithms in individuals carrying the E280A *PSEN1* mutation and individuals with *s*AD. The set of predictor variables consisted of demographic variables (i.e., gender, sex, and years of education) and genomic variants previously identified to be associated as ADAOO modifiers (Table 1). The complete list of ML algorithms is provided in the Supplementary Materials. Construction, parameters tuning, validation, and testing of these predictive models were performed in R version 4.0.2 Patched (2020-06-30 r78761) [80] with the methods implemented in the caret package [47,48] using a 10-fold cross-validation procedure with five repetitions. The training/testing data sets consisted of 70%/30% of individuals per cohort. Given the continuous nature of the outcome variable (i.e., ADAOO), the root mean squared error (RMSE), the R-squared (*R*^2^), and the mean absolute error (MAE) measures were used to evaluate the performance of the ML algorithms. In ML-based predictive models, high values of *R*^2^ and low values of RMSE and MAE indicate good performance. To graphically represent the performance of these ML algorithms and to identify similarities among them, we combined *K*-means clustering [81] and principal component analysis (PCA) [82,83]; the number of *K*-means clusters and the number of principal components were determined using the methods implemented in the NbClust [84] and paran [85] packages for R.

To evaluate the stability of each predictor’s variable importance, we implemented the following resampling strategy, which is a slight modification of the empirical bootstrap [86,87]. First, we constructed *B =* 1000 training data sets at random, keeping the 70%/30% proportion for the training/testing data sets initially used to identify the best performing ML model. Secondly, for the *b*-th training data set (*b* = 1,2, …, *B*), this model was fitted, and the variable importance measure associated with each predictor was computed. Thus, for any predictor *X*, we obtained the values *X*^(1)^, *X*^(2)^, *X*^(3)^, …, *X*^(*B*)^, with *X*^(*b*)^ representing the variable importance of *X* calculated in the *b*-th randomly generated training data set. Finally, we calculated the bootstrap-based 95% confidence intervals (CIs) based on the 2.5% and 97.5% percentiles of *X*^(1)^, *X*^(2)^, *X*^(3)^, …, *X*^(*B*)^.

## 3. Results

### 3.1. ADAOO Prediction in the fAD E280A Pedigree

Table 2 presents the performance measures for ML algorithms’ collection for predicting AOO in the E280A pedigree. The training/testing data sets consisted of 51/20 individuals, respectively. When predicting AOO in the training data set, the xgbLinear ML algorithm outperformed all other algorithms in the RMSE, *R*^2^, and MAE performance measures. When evaluating these ML algorithms’ performance for unseen data (i.e., testing data set), the glmboost ML algorithm outperformed all other alternatives.

**Figure 1:**
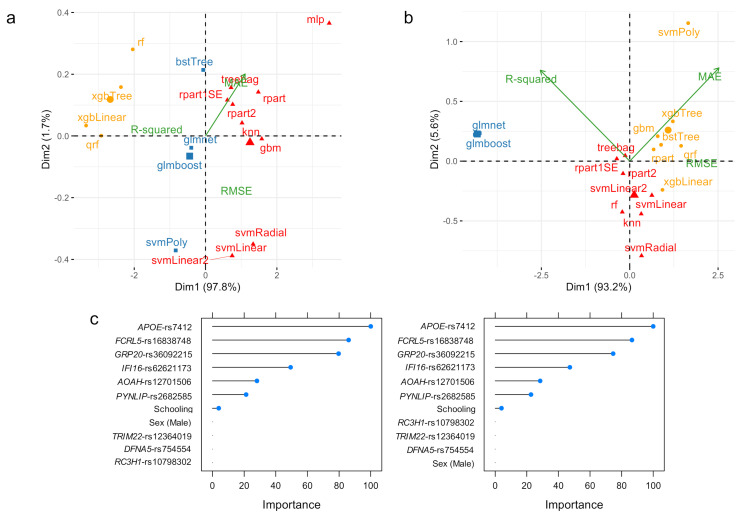
PCA and *K*-means clustering representation of the performance measures for ML algorithms predicting ADAOO in individuals carrying the *PSEN1* E280A mutation when the (**a**) training (*n* = 51) and (**b**) testing (*n* = 20) data sets are used. (**c**) Variable importance for the glmnet (left) and glmboost (right) ML algorithms. Here, higher values are better.

Following our results, the performance of these ML algorithms can be grouped into three classes. For the training data set, class 1 comprises the rf, xgbTree, xbLinear, and qrf algorithms (Figure 1a; yellow); class 2 is constituted by the mlp, treebag, rpart1SE, rpart2, rpart, knn, gbm, svmRadial, svmLinear, and svmLinear2 algorithms (Figure 1a; red); and class 3 by the bstTree, glmnet, glmboost, and svmPoly algorithms (Figure 1a; blue). In the testing data set, the svmPoly, xgbTree, xgbLinear, gbm, bstTree, rpart, and qrf algorithms belong to class 1 (Figure 1b; yellow); tree bag, rpart1SE, rpart2, svmLinear, svmLinear2, rf, knn, and svmRadial form class 2 (Figure 1a; red); and glmnet and glmboost constitute class 3 (Figure 1b; blue). Overall, the best performing algorithms are grouped into class 1 for the training data set, and into class 3 for the testing data set; the xgbLinear algorithm outperforms all other alternatives in class 1 (Table 2 and Figure 1a), while the glmboost algorithm outperforms those in class 3 (Table 2 and Figure 1b).

Figure 1c depicts variable importance plots for the xgbLinear, glmnet, and glmboost algorithms. Our results suggest that, for the xgbLinear algorithm, which is more suitable for assessing ADAOO in the training data set, years of education (Schooling), genetic variants *GPR20*-rs36092215 and *PYNLIP*-rs2682585, and sex (i.e., being male) are the most important predictors of ADAOO (Figure 1c, left). For the glmnet and glmboost algorithms, which outperform the other alternatives when predicting ADAOO for unseen data, the most important predictors are the genetic variants *APOE*-rs7412, *FCRL5*-rs16838748, *GRP20*-rs36092215, *IFI16*-rs62621173, *AOAH*-rs12701506, and *PYNLIP*-rs2682585, followed by years of education (Figure 1c, center; Figure 1c, right).

### 3.2. ADAOO Prediction in the Sporadic AD

Table 3 presents the performance measures for collecting ML algorithms used to predict AOO in individuals of the *s*AD cohort. The training and data sets consisted of 40 and 14 individuals, respectively. When predicting AOO in the training data set, the svmLinear and xgbLinear ML algorithms perform reasonably well, with the latter algorithm outperforming all others in terms of the RMSE, *R*^2^, and MAE performance measures. Despite its remarkable performance in the training data set, the predictive power of the xgbLinear algorithm is rather week in unseen data (i.e., possible overlearning). Thus, the svmLinear algorithm seems to be a better alternative than xgbLinear algorithm. On the other hand, when evaluating the performance of these ML algorithms for the testing data set, the lasso outperforms the other alternatives in terms of the RMSE and *R*^2^, while the glmnet algorithm does so in terms of the MAE (Table 3). In contrast, these ML algorithms are strong learners.

**Figure 2:**
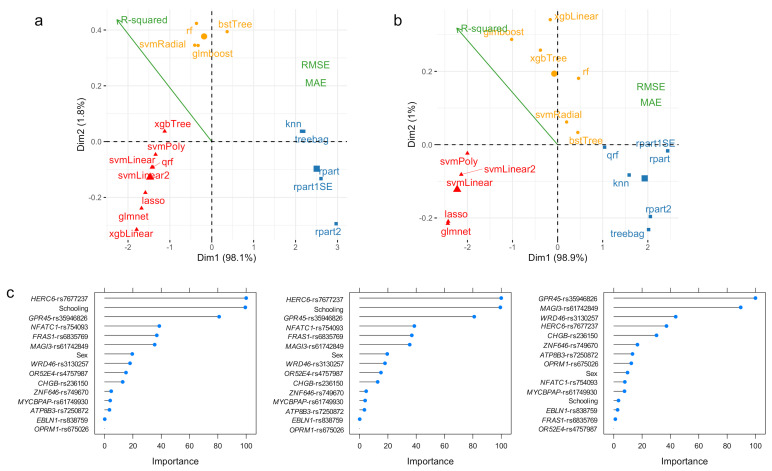
PCA and *K*-means clustering representation of the performance measures for ML algorithms predicting ADAOO in individuals with sporadic AD from the Paisa genetic isolate when the (**a**) training (*n* = 40) and (**b**) testing (*n* = 14) data sets are used. (**c**) Variable importance for the svmLinear (left), lasso (center) and glmnet (right) ML algorithms. Conventions as in Figure 1.

Our results indicate that these ML algorithms’ performance can be grouped into three classes. For the training data set, class 1 comprises the bstTree, glmboost, rf, and svmRadial algorithms (Figure 2a; yellow); class 2 is constituted by the xgbTree, svmPoly, qrf, svmLinear, svmLinear2, lasso, glmnet, and xbgLinear algorithms (Figure 2a; red); and class 3 by the treebag, knn, rpart1SE, rpart, and rpart2 algorithms (Figure 2a; blue). In the testing data set, the glmboost, xgbTree, rf, svmRadial, and bstTree algorithms belong to class 1 (Figure 2b, yellow); svmPoly, svmLinear, svmLinear2, lasso, and glmnet algorithms belong to class 2 (Figure 2b; red); and treebag, rpart, rpart1SE, rpart2, and qrf constitute class 3 (Figure 2b; blue). Overall, the best performing algorithms are grouped into class 2 for both the training and testing data sets; the xgbLinear algorithm outperforms all other alternatives for the training data set (Table 3 and Figure 2a), while the lasso and glmnet algorithms seem to be the best options for unseen data (Table 3 and Figure 2b).

Figure 2c depicts variable importance plots for the svmLinear, lasso, and glmnet algorithms. We identified that for the svmLinear and lasso algorithms, the most important predictors of ADAOO are variants *HERC6*-rs7677237, years of education, *GPR45*-rs35946826, *NFATC1*-rs754093, *FRAS1*-rs6835769 and *MAGI3*-rs61742849, and *CENPJ*-rs17081389 (Figure 2c, left and Figure 2c, center). Interestingly, under the svmLinear and lasso ML algorithms, sex is a seemingly significant predictor of ADAOO. In terms of variable importance, the glmnet ML algorithm yields similar results to those in the svmLinear and lasso algorithms, but highlights the relevance of variants *GPR45*-rs35946826, *MAGI3*-rs61742849, *C16orf96*-rs17137138, and *C3orf20*-rs34230332, and the small contribution to ADAOO of sex and years of education in unseen individuals with *s*AD (Figure 2c, right).

### 3.3. Variable Importance: Stability and Relationship with $\hat{\beta }$

**Figure 3:**
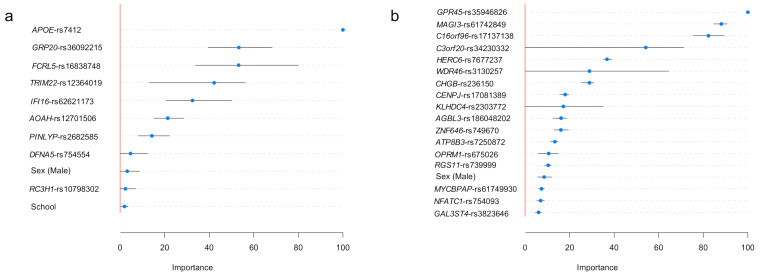
Variable importance for the best ADAOO-predicting ML algorithm in individuals (**a**) carrying the E280A mutation and (**b**) individuals with *s*AD. Blue dots represent the average importance; segments represent 95% bootstrap-based confidence intervals based on *B =* 1000 replicates. Conventions as in Figure 1.

Figure 3 shows our implementation results for evaluating variable importance stability for each predictor in the best ML algorithm. When predicting ADAOO in individuals carrying the E280A mutation, the most important predictor is, by far, the *APOE*-rs7412 genetic variant, and the least essential predictors are sex, the genetic variant *RC3H1*-rs10798302, and years of education (Figure 3a).

**Figure 4:**
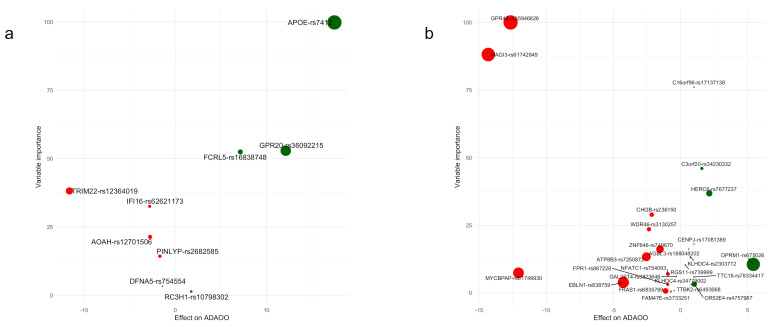
Variable importance vs. effect on ADAOO for genetic variants in individuals with (**a**) E280A *PSEN1* and (**b**) sporadic AD. Protective ($\hat{\beta }$ > 0) variants are shown in green, while harmful ($\hat{\beta }$ < 0) variants are shown in red. See Table 1 for more details.

In individuals with *s*AD, the most important ADAOO predictor is the genetic variant *GPR45*-rs359446826, followed by variants *MAGI3*-rs61742849, *C16orf96*-rs17137138, and *C3orf20*-rs34230332. Interestingly, sex and years of education (not shown) are among the least important predictors (Figure 3b). Variable importance bootstrap-based distributions are provided in Figures S1 and S2 (Supplementary Materials). Figure 4 shows scatterplots between $\hat{\beta }$ and their variable importance predicting ADAOO (Table 2 and Table 3), confirming that, in contrast to *f*AD, essential predictors of ADAOO in *s*AD correspond to several genetic variants of small effect [29,30].

## 4. Discussion

Machine learning (ML) algorithms have recently caught the scientific community’s attention because of their flexibility, ease of use, and ability to learn from the data provided [55,56]. Via ML, it has been possible to develop models to identify individuals more susceptible to developing common and rare diseases [58,59,60,61,62,63,67,88,89,90,91,92,93] and determine diverse phenotypic response profiles in infectious diseases [94,95,96]. Considering that ML- and computational-based models have the potential to overcome the limitations of current established clinical models for the diagnosis and follow-up of neurodegenerative diseases, including AD [97], here we studied the feasibility of ML algorithms for predicting Alzheimer’s disease age of onset (ADAOO) in individuals from the Paisa genetic isolate. We argue that these ML-based predictive models will improve our understanding of the disease and provide a more accurate and precise definition of the AD natural history landmarks.

We previously identified protective ($\hat{\beta }\;$> 0; Table 1) and harmful ($\hat{\beta }\;$< 0; Table 1) ADAOO-modifying variants of significant effect in this community from whole-exome genotyping and whole-exome sequencing data [35,36] using linear-mixed effects models and some ML methods [77]. Thus, the presence of the APOE*E2 allele alone delays ADAOO up to ~12 years in *PSEN1* E280A mutation carriers. Furthermore, this same allele delays ADAOO up to ~17 years when included in an AD oligogenic model (Table 1) [36]. Subsequent analysis led to the development of a classification tree using advanced recursive partitioning to determine whether individuals carrying this mutation would develop early-onset or late-onset familial AD [36]. Following a similar approach, our group was able to identify ADAOO modifier variants in individuals with sporadic AD (Table 1) [35].

After evaluating several ML-based predictive algorithms for ADAOO in individuals suffering from the most aggressive form of AD (Figure 1 and Table 2) and in individuals with sporadic AD (Figure 2 and Table 3), we identified that the glmboost and glmnet algorithms perform best for predicting ADAOO in unseen data for each cohort, respectively. These ML-based predictive models showed promising results that can be easily extended to the clinical setting [98]. In particular, the glmboost algorithm in E280A *PSEN1* AD yielded MAE values below 4% and RMSE values of ~4 (Table 2), while the glmnet algorithm yielded MAE values below 1% and RMSE values <1 in *s*AD (Table 3), suggesting that predicting AOO in these cohorts is feasible. Using these ML-based ADAOO predictive models, AD diagnosis could be made earlier, and potential treatments are provided long before symptoms begin to appear.

Analysis of variable importance shows that the most relevant ADAOO predictors in *f*AD are variants *APOE*-rs7412, *FCRL5*-rs16838748, *GPR20*-rs36092215, *IFI16*-rs62621173, *AOAH*-rs12701506, and *PYNLIP*-rs2682585 (Figure 1b and Figure 3a). Furthermore, protective variants *APOE*-rs7412, *GRP20*-rs36092215, and *FCRL5*-rs16838748 have both the highest effect on ADAOO and are the most important predictors of ADAOO, while variants *TRIM22*-rs12364019, *IFI16*-rs62621173, and *AOAH*-rs12701506 have both the most harmful effect on ADAOO and are among the most important predictors of ADAOO (Figure 4a). Comparing these results with those of previous models predicting AD status (early- vs. late-onset) [36] shows some discrepancies in how the genetic variants are ranked and the relevance of demographic information (i.e., sex and years of education) for predicting AD status. Although predicting AD status may be of interest in some clinical settings, the use of ML-based predictive algorithms for ADAOO is a step forward in both our understanding of the disease and our goal of providing timely clinical care to individuals from this community. While AD cannot be cured and there is no way to stop or slow its progression at the moment, our approach offers the possibility of treating symptoms several years before they begin to appear [4,99,100] under an individually tailored biomarker scheme rather, than using a one-size-fits-all population average strategy [99,100,101], while taking individual variability into account. Although our results can certainly be used to move AD research in this direction, it is also important to consider the legal implications and the preparation that health providers, neurologists, and centers specializing in AD and neurodegeneration must have in order to interpret these findings and provide proper counseling to patients and their families [102,103,104]. Another challenge in the years to come is also to significantly reduce the misinformed conclusions produced by ML methods in the absence of clinical domain expertise [105]. In this regard, having a deep understanding of the clinical background in AD, how ML methods operate, and how the results can interpreted and translated to the patient and their relatives is crucial [57].

Variants *GPR45*-rs35946826 and *MAGI3*-rs61742849 have both a more harmful effect on ADAOO and are the most important predictors of ADAOO in individuals with *s*AD (Figure 4b). Interestingly, the harmful effect on ADAOO of variants *MYCBPAP*-rs61749930 and *EBLN1*-rs838759 differs from those of other variants, but their importance for predicting ADAOO is lower, while variants *CHGB*-rs236150 and *WDR46*-rs3130257 accelerate ADAOO and have higher variable importance (Figure 4b). Among protective genetic variants, the highest effect is produced by *OPRM1*-rs675026, followed by *HERC6*-rs7677237 and *C3orf20*-rs34230332, with the former being the less important. Intriguingly, variant *C16orf96*-rs17137138 is the most important ADAOO predictor despite its small effect (Figure 4b).

In summary, here we explore the feasibility of ML algorithms for predicting ADAOO using demographic and genetic data in individuals from the world’s most extensive pedigree segregating a severe form of AD caused by a fully penetrant mutation in the *PSEN1* gene and individuals with *s*AD inhabiting the same geographical region. Based on the RMSE, MAE, and *R*^2^ performance measures, our results indicate that ML algorithms are a feasible and promising alternative for assessing ADAOO in these individuals. Interestingly, the most important predictors in these ML-based predictive models were genetic variants, which makes it possible to assess ADAOO at the individual level and opens new personalized medicine and predictive genomic alternatives for AD [98,99,100,101].

Future studies should assess the ability of the ML-based predictive models for ADAOO presented herein with out-of-sample data (i.e., determine how close the model is to predicting ADAOO in a patient with known genetic data that was not part of our cohorts) and the development of ML-based models of disease progression [38,50,51,60]. Ultimately, these models could help us to provide an easy-to-use platform, with potential application in the clinical setting, to provide early and accurate estimates of ADAOO and the evolution of AD in individuals with a family history of the disease.

## Figures and Tables

**Table 1:** Common exonic functional variants modifying ADAOO in 125 individuals from the Paisa genetic isolate.

Cohort	Chr	Marker	Position *^a^*	Gene	Change	$\hat{\beta }$ $(SE_{\hat{\beta }})\;$ *^b^*	*P*_FDR_
E280A	19	rs7412	45,412,079	*APOE*	p.Arg176Cys	17.45 (0.48)	2.13 × 10^−30^
(*n* = 71)	8	rs36092215	142,367,246	*GPR20*	p.Arg260Cys	12.12 (0.54)	6.58 × 10^−22^
	11	rs12364019	5,730,343	*TRIM22*	p.Arg321Lys	−11.64 (0.79)	1.15 × 10^−14^
	1	rs16838748	157,508,997	*FCRL5*	p.Asn427Lys	7.14 (0.68)	8.61 × 10^−10^
	7	rs12701506	36,566,020	*AOAH*	*^c^*	−2.75 (0.30)	5.69 × 10^−8^
	19	rs2682585	44,081,288	*PINLYP*	p.His6Arg	−1.68 (0.21)	1.67 × 10^−6^
	1	rs62621173	159,021,506	*IFI16*	p.Ser512Phe	−2.80 (0.37)	8.63 × 10^−6^
	1	rs10798302	173,987,798	*RC3H1*	*^d^*	1.76 (0.27)	1.86 × 10^−4^
	7	rs754554	24,758,818	*DFNA5*	p.Pro142Thr	−1.39 (0.28)	3.62 × 10^−2^
Sporadic	2	rs35946826	105,859,249	*GPR45*	p.Leu312fs	−12.67 (0.148)	3.08 × 10^−36^
(*n* = 54)	1	rs61742849	114,226,143	*MAGI3*	p.Gly1318fs	−14.32 (0.199)	4.38 × 10^−34^
	6	rs675026	154,414,563	*OPRM1*	p.Ala442fs	5.42 (0.079)	1.15 × 10^−33^
	10	rs838759	22,498,468	*EBLN1*	p.Gly149fs	−4.26 (0.092)	3.90 × 10^−28^
	17	rs61749930	48,594,691	*MYCBPAP*	p.Arg124fs	−12.08 (0.286)	6.06 × 10^−27^
	19	rs7250872	1,811,603	*ATP8B3*	p.Gly45fs	−2.54 (0.088)	9.57 × 10^−22^
	16	rs749670	31,088,625	*ZNF646*	p.Lys328fs	−1.52 (0.067)	1.35 × 10^−18^
	4	rs7677237	89,306,659	*HERC6*	p.Met123fs	2.14 (0.122)	3.58 × 10^−15^
	4	rs6835769	79,284,694	*FRAS1*	p.Ala817fs	−1.11 (0.074)	2.74 × 10^−13^
	11	rs4757987	5,906,205	*OR52E4*	p.Arg228fs	1.02 (0.07)	6.86 × 10^−13^
	20	rs236150	5,903,141	*CHGB*	p.Lys117fs	−2.14 (0.181)	2.12 × 10^−10^
	6	rs3130257	33,256,471	*WDR46*	p.Thr40fs	−2.35 (0.209)	7.92 × 10^−10^
	18	rs754093	77,246,406	*NFATC1*	p.Cys751fs	−0.94 (0.094)	1.34 × 10^−8^
	3	rs34230332	14,725,878	*C3orf20*	p.Leu84fs	1.59 (0.185)	4.81 × 10^−7^
	19	rs867228	52,249,211	*FPR1*	p.Glu346fs	−0.94 (0.115)	1.34 × 10^−6^
	4	rs3733251	77,192,838	*FAM47E*	p.Arg166fs	−0.71 (0.127)	2.07 × 10^−3^
	16	rs2303772	87,795,580	*KLHDC4*	p.Leu56fs	0.75 (0.135)	2.75 × 10^−3^
	16	rs739999	319,511	*RGS11*	p.Met416fs	0.35 (0.075)	3.48 × 10^−2^
	16	rs34779002	87,782,396	*KLHDC4*	p.Gly74fs	0.78 (0.172)	4.00 × 10^−2^
	15	rs6493068	43,170,793	*TTBK2*	p.Asp9fs	−0.48 (0.107)	4.27 × 10^−2^
	16	rs17137138	4,606,743	*C16orf96*	p.Val85fs	1.00 (0.223)	4.40 × 10^−2^
	7	rs3823646	99,757,612	*GAL3ST4*	p.Lys468fs	−0.31 (0.069)	4.47 × 10^−2^
	13	rs17081389	25,487,001	*CENPJ*	p.Pro55fs	1.00 (0.223)	4.61 × 10^−2^
	10	rs78334417	75,071,618	*TTC18*	p.Pro450fs	1.00 (0.223)	4.84 × 10^−2^
	7	rs186048202	134,678,273	*AGBL3*	p.Arg52fs	0.61 (0.139)	4.91 × 10^−2^

*^a^* UCSC GRCh37/hg19 coordinates; *^b^* Markers can accelerate ($\hat{\beta }$ < 0) or delay ($\hat{\beta }\;$> 0) ADAOO according to their effect; *^c^* Chromatin state segmentation strong enhancer state-5 from ChiP-seq data; *^d^* CpG islands, DNaseI hypersensitivity uniform peak from ENCODE/analysis. ADAOO = Alzheimer’s disease age of onset; Chr: chromosome; $\hat{\beta }$ = Regression coefficient; ${\mathrm{SE}}_{\hat{\beta }}$ = Standard error of $\hat{\beta }$; *P*_FDR_ = Corrected *P*-value using the False Discovery Rate (FDR) [78,79].

**Table 2:** Performance of ML algorithms for predicting ADAOO in the E280A pedigree. RMSE = root mean squared error, lower is better; MAE = mean absolute error, lower is better; *R*^2^ = coefficient of determination, higher is better. Best results are shown in **bold**.

ML Algorithm	Performance Measure					
	RMSE		*R* ^2^		MAE	
	Training	Testing	Training	Testing	Training	Testing
glmboost	3.51	**3.73**	0.62	**0.65**	2.41	**2.86**
bstTree	3.67	6.75	0.59	0.08	3.00	4.52
gbm	4.90	6.68	0.27	0.09	3.86	4.52
glmnet	3.59	3.85	0.62	0.64	2.51	2.89
knn	4.53	6.35	0.39	0.05	3.56	4.13
mlp	6.30	6.62	0.07	0.43	5.64	5.78
qrf	1.35	7.24	0.95	0.03	0.69	4.65
rf	2.14	6.17	0.91	0.12	1.70	3.93
rpart	4.73	6.36	0.31	0.07	3.95	4.51
rpart1SE	4.18	5.89	0.46	0.18	3.35	4.11
rpart2	4.28	6.02	0.43	0.15	3.43	4.11
svmLinear	4.74	6.80	0.43	0.07	2.97	4.21
svmLinear2	4.74	6.80	0.43	0.07	2.97	4.21
svmPoly	3.46	7.30	0.66	0.14	1.86	5.13
svmRadial	5.21	6.50	0.35	0.02	3.43	3.96
treebag	4.26	6.02	0.45	0.16	3.47	4.20
xgbLinear	**0.85**	7.14	**0.98**	0.06	**0.37**	4.28
xgbTree	1.79	7.12	0.90	0.08	1.28	4.65

**Table 3:** Performance of ML algorithms for predicting ADAOO in the individuals with sporadic AD from the Paisa genetic isolate. Conventions as in Table 2. Best results are shown in **bold**.

ML Algorithm	Performance Measure					
	RMSE		*R* ^2^		MAE	
	Training	Testing	Training	Testing	Training	Testing
bstTree	3.33	5.22	0.83	0.44	2.56	3.75
glmboost	2.32	3.08	0.92	0.84	1.96	2.47
glmnet	0.25	0.52	1.00	0.99	0.17	**0.39**
knn	5.37	6.75	0.48	0.16	3.90	4.98
lasso	0.40	**0.52**	1.00	**1.00**	0.31	0.42
qrf	0.87	5.86	0.99	0.30	0.40	4.57
rf	2.47	5.09	0.94	0.49	1.86	4.15
rpart	5.53	7.69	0.38	0.00	4.46	6.37
rpart1SE	5.53	7.69	0.38	0.00	4.46	6.37
rpart2	5.92	6.98	0.29	0.03	4.63	5.75
svmLinear	0.61	1.11	0.99	0.97	0.57	0.83
svmLinear2	0.61	1.11	0.99	0.97	0.57	0.83
svmPoly	0.75	1.33	0.99	0.96	0.70	1.07
svmRadial	2.57	4.70	0.93	0.51	1.57	3.64
treebag	5.22	7.02	0.48	0.02	4.13	5.54
xgbLinear	**0.03**	4.61	**1.00**	0.67	**0.02**	3.32
xgbTree	1.13	3.98	0.98	0.70	0.93	3.19

## Data Availability

Not applicable.

## Supplementary Materials

The following are available online at https://www.mdpi.com/article/10.3390/diagnostics11050887/s1, Table S1: ML algorithms used to predict ADAOO; Figure S1: Variable importance bootstrap-based density distribution for ADAOO predictors in E280A AD; Figure S2: Variable importance bootstrap-based density distribution for ADAOO predictors in *s*AD.
