# Supplementary material for: A Comprehensive Machine Learning Framework for the Exact Prediction of the Age of Onset in Familial and Sporadic Alzheimer’s Disease
Source: Diagnostics (Basel). 2021 May 17;11(5):887. doi: 10.3390/diagnostics11050887 (PMC8156402; doi:10.3390/diagnostics11050887)
Supplement: Supplementary file 1 [file diagnostics-11-00887-s001.zip › diagnostics-1192249-supplementary.pdf]

diagnostics

Supplementary Material

Predictive Genomics Models of Alzheimer's disease age of onset

We used Machine Learning (ML) algorithms to construct predictive models of Alzheimer's disease age of onset (ADAOO) as implemented in the R caret package (Kunh, 2020). Table S1 shows the ML algorithms used in this study.[1] In all cases, the set of predictors for ADHD severity consisted of demographic variables, genetic markers and ADHD affection status. See the Methods section in the main manuscript for more details.

Table S1. ML algorithms used to predict ADAOO.

Method	Model	Tuning parameters
avNNet	Model Averaged Neural Network	size, decay, bag
bstTree	Boosted Tree	mstop, maxdepth, nu
gbm	Stochastic Gradient Boosting	n.trees, interaction.depth, shrinkage, n.minobsinnode
glmboost	Boosted Generalized Linear Model	mstop, prune
glmnet	glmnet	alpha, lambda
knn	k-Nearest Neighbors	k
lasso	The lasso	fraction
lda	Linear discriminant analysis	None
mlp	Multi-Layer Perceptron	size
rf	Random Forest	mtry
rpart	CART	cp
rpart1SE	CART	None
rpart2	CART	maxdepth
svmLinear	SVM with Linear Kernel	C
svmLinear2	SVM with Linear Kernel	Cost
svmPoly	SVM with Polynomial kernel	degree, scale, C
svmRadial	SVM with Radial Basis Function Kernel	sigma, C
treebag	Bagged CART	None
xgbLinear	eXtreme Gradient Boosting (XGBoost)	nrounds, lambda, alpha, eta
xgbTree	eXtreme Gradient Boosting Tree	nrounds, max_depth, eta, gamma, colsample_bytree, min_child_weight, subsample

CART: Classification and Regression Tree (CART); (L. Breiman, Friedman, Olshen, & Stone, 1984) RF: Random Forest (RF);(L. Breiman, 2001; Satterfield, Cantwell, & Satterfield) SVM: Support Vector Machine (SVM);(Cortes & Vapnik, 1995; Salazar, Vélez, & Salazar, 2012) XGBoost: eXtreme Gradient Boosting.(Chen & Guestrin, 2016; Chen et al., 2020).

[1] The complete list of ML algorithms implemented in caret is available at https://topepo.github.io/caret/available-models.html

diagnostics

MDPI

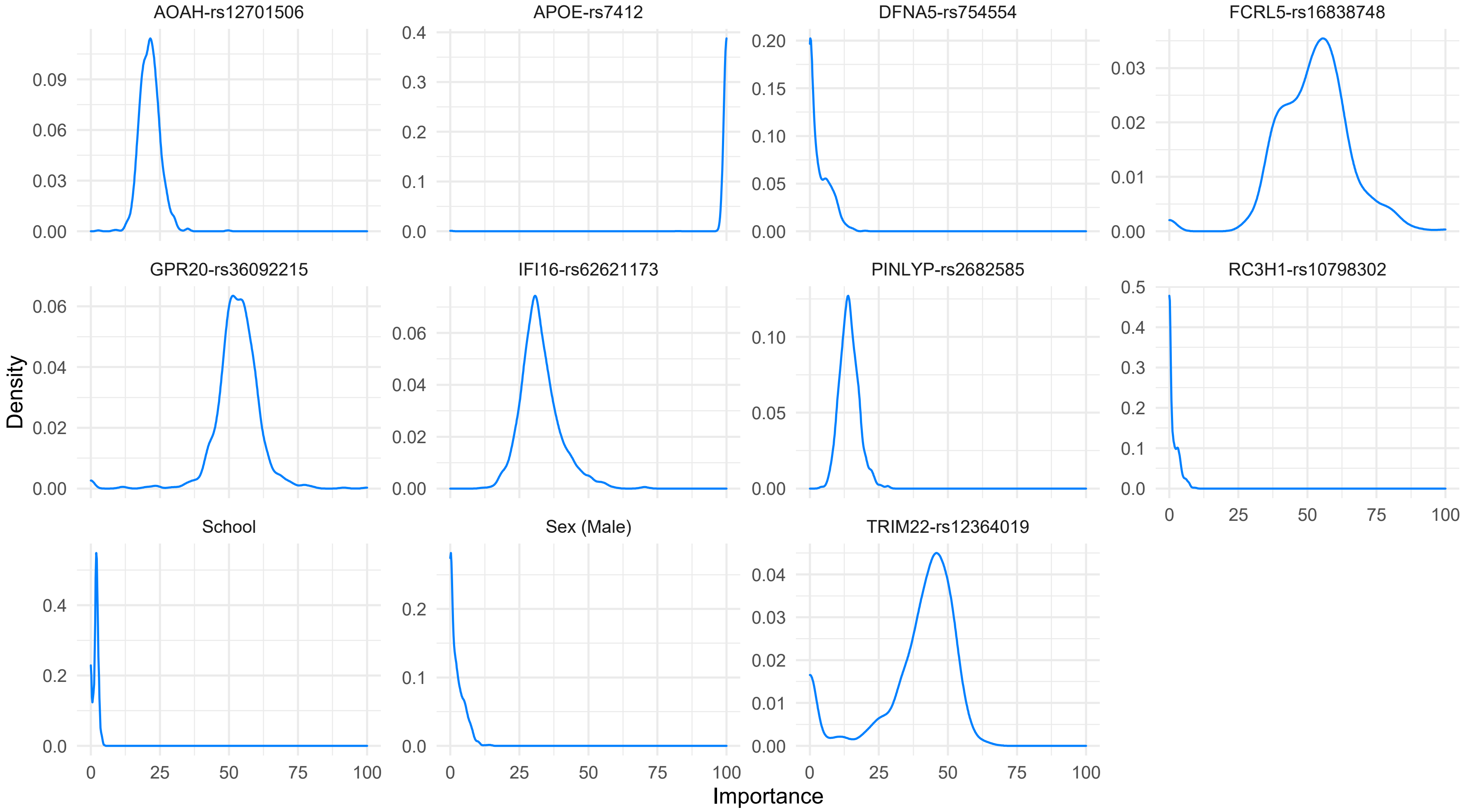

Figure S1. Variable importance bootstrap-based density distribution for ADAOO predictors in E280A AD using the glmboost ML algorithm.

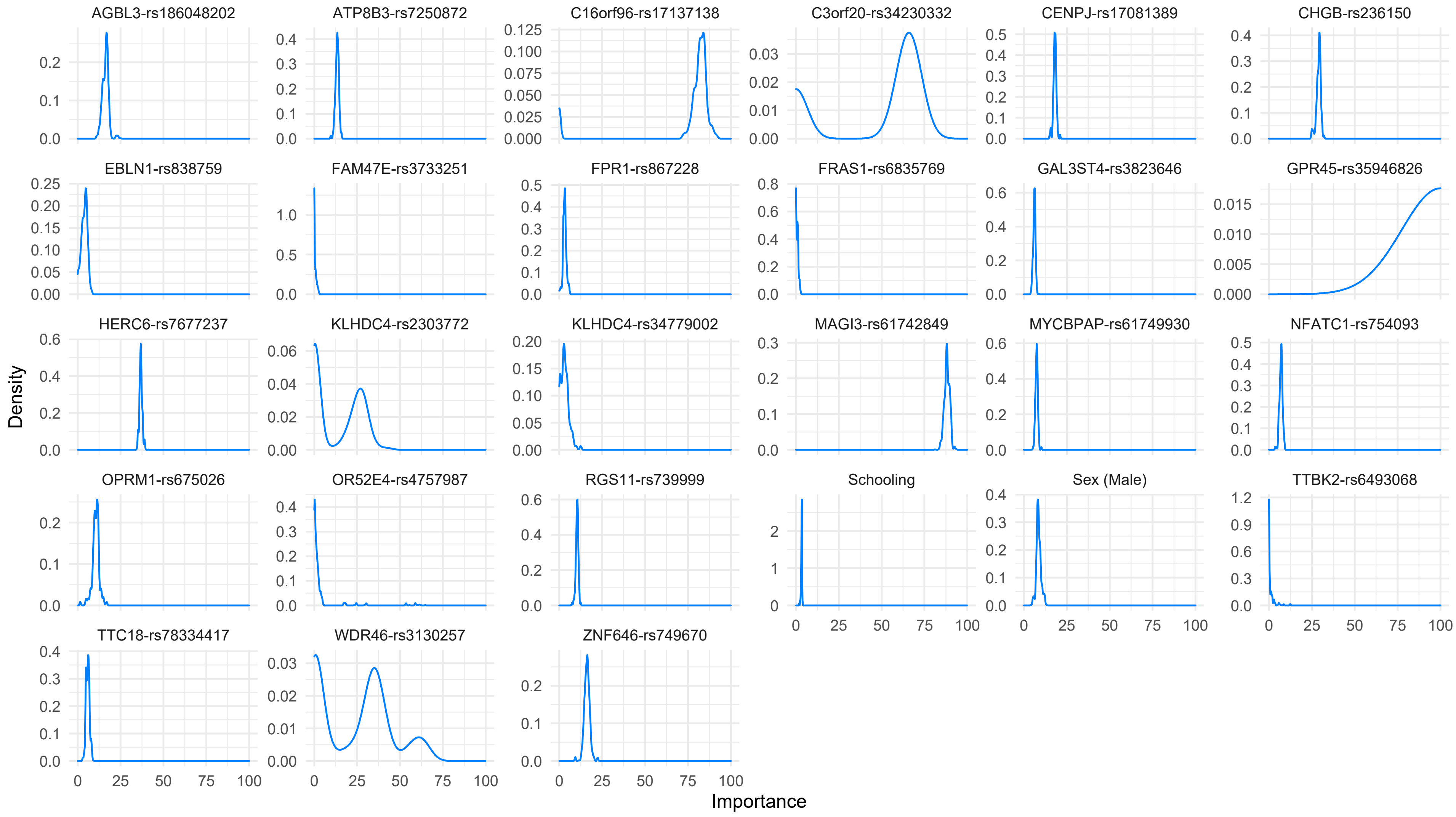

Figure S2. Variable importance bootstrap-based density distribution for ADAOO predictors in sAD using the glmnet ML algorithm.

References

1. Breiman, L. (2001). Random Forests. In R. E. Schapire (Ed.), *Machine Learning* (Vol. 45, pp. 5-32). Statistics Department, University of California, Berkeley, CA 94720: Kluwer Academic Publishers. Manufactured in The Netherlands.
2. Breiman, L., Friedman, J. H., Olshen, R. A., & Stone, C. H. (1984). *Classification and Regression Trees.* Belmont, CA: Wadsworth International Group, Inc.
3. Chen, T., & Guestrin, C. (2016). *XGBoost: A Scalable Tree Boosting System*. Paper presented at the 22nd SIGKDD Conference on Knowledge Discovery and Data Mining. https://arxiv.org/abs/1603.02754
4. Chen, T., He, T., Benesty, M., Khotilovich, V., Tang, Y., Cho, H., . . . Li, Y. (2020). xgboost: Extreme Gradient Boosting. R package version 1.0.0.2. URL: https://CRAN.R-project.org/package=xgboost.
5. Cortes, C., & Vapnik, V. (1995). Support-vector networks. *Machine Learning, 20*, 273-297. doi:https://doi.org/10.1007/BF00994018
6. Kunh, M. (2020). caret: Classification and Regression Training. (Version R package version 6.0-86). Retrieved from https://CRAN.R-project.org/package=caret
7. Salazar, D. A., Vélez, J. I., & Salazar, J. C. (2012). Comparison between SVM and Logistic Regression: Which one is Better to Discriminate? *Revista Colombiana de Estadistica, 35*(2), 223-237.
8. Satterfield, J. H., Cantwell, D. P., & Satterfield, B. T. (1974). Pathophysiology of the hyperactive child syndrome. *Arch Gen Psychiatry, 31*(6), 839-844. Retrieved from https://www.ncbi.nlm.nih.gov/pubmed/4441251.
